# Supplementary material for: Prot2Chat: protein large language model with early fusion of text, sequence, and structure
Source: Bioinformatics. 2025 Jul 28;41(8):btaf396. doi: 10.1093/bioinformatics/btaf396 (PMC12341678; doi:10.1093/bioinformatics/btaf396)
Supplement: btaf396_Supplementary_Data [file btaf396_supplementary_data.pdf]

Appendix of ”Prot2Chat: Protein LLM with Early-Fusion of Text, Sequence and Structure”

Zhicong Wang[1], Zicheng Ma[2], Ziqiang Cao[*1], Changlong Zhou[1], Jun Zhang[*2], Yiqin Gao[2]

[1]*School of Computer Science and Technology, Soochow University, Suzhou, 215006, China*
[2]*Changping Laboratory, Beijing, 102206, China*

Appendix Introduction

This appendix provides supplementary materials for the paper ”Prot2Chat: Protein LLM with Early-Fusion of Text, Sequence and Structure”. Its purpose is to offer some details, evaluation methods, and practical guidelines to complement the main text, enabling readers to comprehensively understand the implementation and performance of the model.

The appendix is mainly divided into three parts. Section 1 elaborates on the evaluation metrics used to assess Prot2Chat. These include traditional natural language processing metrics (BLEU (Papineni et al., 2002), ROUGE (Lin, 2004)), as well as new methods such as online large language model-assisted evaluation and expert manual evaluation. These metrics cover aspects such as lexical-syntactic alignment and high-level semantic understanding, ensuring a comprehensive analysis of the model’s performance.

Section 2 provides a step-by-step GitHub project tutorial, guiding users through steps such as environment setup, model preparation, data input, and web deployment. This practical guide aims to promote the reproducibility of the research and enable the research community to explore and expand the Prot2Chat framework.

Finally, section 3 presents more case studies. These studies demonstrate the model’s capabilities in predicting protein subcellular localization, identifying motifs/domains, and inferring biological functions. These examples highlight the advantages of Prot2Chat over sequence-only models, showcasing its effectiveness in integrating structure, sequence, and text information.

Overall, these appendices form a supplementary resource, providing theoretical details of evaluation methods as well as practical instructions for model implementation, thus enhancing the reproducibility and practicality of this research.

1 Details on Evaluation Metrics

In our implementation, we use different metrics for the proposed Prot2Chat to verify its performance. These include traditional natural language processing metrics such as BLEU and ROUGE, as well as new methods like online large language model-assisted evaluation and expert manual evaluation. These metrics focus not only on lexical and syntactic alignment but also on high-level semantic information.

1.1 BLEU

BLEU (Papineni et al., 2002) (BiLingual Evaluation Understudy) serves as a crucial metric for assessing the quality of the machine-generated text, particularly in machine translation contexts. It quantifies the similarity between the candidate and

*Corresponding author. Email: zqcao@suda.edu.cn
*Corresponding author. Email: jzhang@cpl.ac.cn

reference text, yielding a score within the range of 0 to 1. A higher BLEU score indicates a closer match between the candidate and reference texts. BLEU is mathematically defined as follows:

$$p_n = \frac{\sum_{C\in\{Candidates\}} \sum_{n\text{-gram}\in C} Count_{clip}(n\text{-gram})}{\sum_{C'\in\{Candidates\}} \sum_{n\text{-gram}'\in C'} Count(n\text{-gram}')} \tag{1}$$

$$\text{BP} = \begin{cases} 1 & \text{if } c > r \\ e^{(1-r/c)} & \text{if } c \leq r \end{cases} \tag{2}$$

$$\text{BLEU} = \text{BP} \cdot exp\left(\sum_{n=1}^{N} w_n log p_n\right) \tag{3}$$

where BP (Brevity Penalty) helps penalize overly short translations and p_n represents the precision of n-grams, ranging from 1 to a predefined maximum.

1.2 ROUGE

ROUGE-N (Lin, 2004) is a fundamental metric extensively employed in the automatic evaluation of text summarization and machine translation. It measures the similarity between a candidate text and one or more reference texts based on the overlap of n-grams (contiguous sequences of n words). Specifically, ROUGE-N computes the recall of n-grams in the candidate text that appear in the reference text(s), emphasizing the comprehensiveness of content coverage in the generated text. The formal definition of ROUGE-N is as follows:

$$R_n = \frac{\sum_{S\in Refs} \sum_{\text{n-gram}\in S} Count_{\text{match}}(\text{n-gram})}{\sum_{S\in Refs} \sum_{\text{n-gram}\in S} Count(\text{n-gram})} \tag{4}$$

$$P_n = \frac{\sum_{S\in Refs} \sum_{\text{n-gram}\in S} Count_{\text{match}}(\text{n-gram})}{\sum_{\text{n-gram}'\in C} Count(\text{n-gram}')} \tag{5}$$

$$\text{ROUGE-N} = \frac{(1+\beta^2)R_n P_n}{R_n + \beta^2 P_n} \tag{6}$$

where: $Refs$ denotes the set of reference texts, and C represents the candidate text. R_n measures n-gram Recall, defined as the ratio of overlapping n-grams (found in both C and any reference in $Refs$) to the total n-grams in all reference texts. P_n measures n-gram Precision, calculated as the ratio of overlapping n-grams to the total n-grams in C. $Count_{\text{match}}(\text{n-gram})$ counts the maximum co-occurrences of an n-gram in C and a specific reference (handling multiple references). The hyperparameter β controls the Recall/Precision balance in the F-score calculation; large β prioritizes Recall (R_n), which is standard for tasks like summarization. ROUGE-N combines R_n and P_n into an F-score, with common variants including ROUGE-1 (unigrams), ROUGE-2 (bigrams), and ROUGE-3 (trigrams) for granular lexical overlap analysis.

ROUGE-L (Lin, 2004) finds frequent use in the automatic evaluation of text summarization and machine translation. It calculates the longest common subsequence between the candidate and reference texts, prioritizing recall over precision. ROUGE-L can be formulated as:

$$R_{lcs} = \frac{LCS(X,Y)}{m} \tag{7}$$

$$P_{lcs} = \frac{LCS(X,Y)}{n} \tag{8}$$

$$\text{ROUGE - L} = \frac{(1+\beta^2)R_{lcs}P_{lcs}}{R_{lcs} + \beta^2 P_{lcs}} \tag{9}$$

where X represents the predicted text with a length of n. Y represents the ground truth with a length of m. β is a hyperparameter used to adjust the emphasis on precision and recall. LCS calculates the length of the longest common subsequence, R_{lcs} measures recall, and P_{lcs} measures precision, respectively.

1.3 Online LLM-assisted Evaluation

Online large language models are built based on deep-learning technologies and possess powerful natural-language understanding and generation capabilities. Their core competencies, such as in-depth parsing of semantics, logic, and context, enable them to serve as effective tools for evaluating the quality of text generation. When presented with a target text and multiple generated texts, they can, through dimensions like semantic similarity calculation, logical coherence judgment, and language normativity verification, accurately screen out the text with the best quality and the highest degree of conformity to the target. In Prot2Chat, we selected the online KIMI (Qin et al., 2024) model as our auxiliary evaluation model. Specifically, the target text and the texts generated by different models are simultaneously input into the online KIMI model, using the following prompts:
Please select the sentence that is closest and most accurate in meaning to the Target sentence and rank sentences A, B, C and D accordingly, with the first place meaning the most identical and accurate in meaning to the Target. Only provide the answer, for example, 'B D A C' or 'A C D B', etc. Target: {Ground Truth} A: {Model1 Generated Text} B: {Model2 Generated Text} C: {Model3 Generated Text} D: {Model4 Generated Text}.

This allowed us to determine which generated answer was closer to the target text, thereby assisting in the evaluation of the model's output quality. Furthermore, we conducted expert manual evaluations, where professional biology PhDs ranked the responses of different models based on their alignment with the target text.

1.4 Expert Manual Evaluation

In order to further ensure the quality of model evaluation, we conducted expert manual evaluations, where professional biology PhDs ranked the responses of different models based on their alignment with the target text.

2 GitHub Project Tutorial

Prot2Chat is a protein Q&A system that allows users to obtain information about proteins through an interactive Q&A mode on the web page. For the related paper, refer to here.

2.1 Environment Installation

2.1.1 Create a Virtual Environment

Use the following commands to create and activate a virtual environment named 'prot2chat':

```
conda create -n prot2chat python=3.9
conda activate prot2chat
```

Then clone the project:

```
git clone https://github.com/wangzc1233/Prot2Chat.git
```

2.1.2 Install Dependencies

Replace '/your/path/to/prot2chat' with the path to your own 'prot2chat' directory, and then run the following commands:

```
cd /your/path/to/prot2chat
bash requirement.sh
```

2.2 Prepare the Models

Download the base model weights from here; download the new adapter weights and LoRA weights from here (this link also includes the modified ProteinMPNN (Dauparas et al., 2022) we used).

Make sure to place the downloaded model and weight files in the specified folders. For example, put ProteinMPNN in the ‘prot2chat/ProteinMPNN‘ directory.

2.3 Prepare Input Data

We have provided several PDB file samples (providing protein structure and sequence information) in the ‘/pdbs‘ directory and some Q&A samples in the ‘/QA_data‘ directory. You can also use your own protein files and Q&A data.

2.4 Run Prot2Chat on the Web

2.4.1 Use ‘demo.py‘

Replace ‘/your/path/to/prot2chat‘ with the path to your own ‘prot2chat‘ directory, and then run the following command:

```
cd /your/path/to/prot2chat
python demo.py --model_path your_path_to_base_model_weight --lora_path
    your_path_to_lora_weight --adapter_path your_path_to_adapter_weight --port
    your_port_ID --gpu your_GPU_IDs
```

After a successful run, access ‘http://localhost:your_port_ID/‘ in your local browser. Select a protein PDB file and enter your question to get an answer, as shown in the Figure 1:

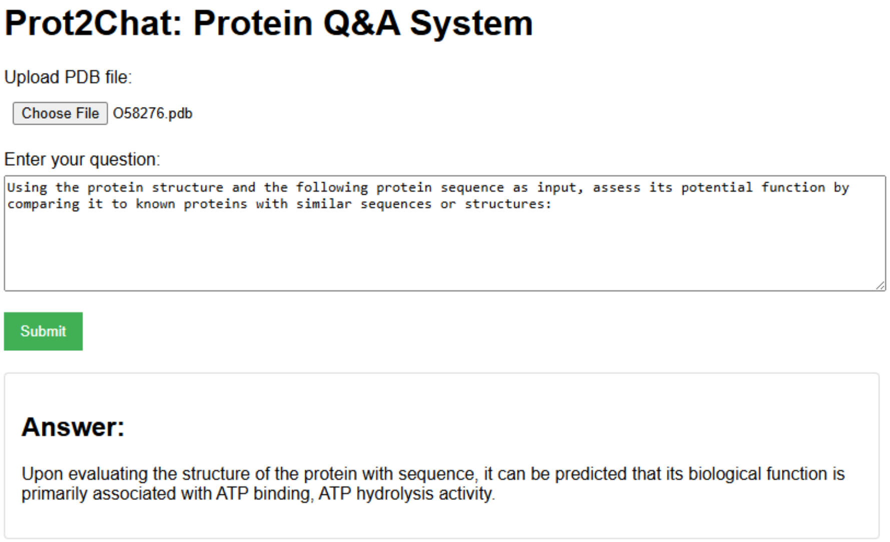

Figure 1: Select a file and enter a question to get an answer

3 More Case Study

To demonstrate the effectiveness of the method architecture of the Prot2Chat model, we have prepared more case studies for reference, as shown in Table 1 and Table 2.

Table 1: More case study. The bold font represents the key points of the answers, the blue font represents the correct answers of the key points, and the red font represents the incorrect answers of the key points.

Inputs	LLaMA3-FT with sequence	Prot2Chat	Ground Truth
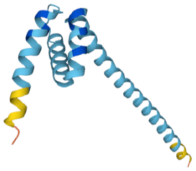 **[O58787]**Using the provided structure and amino acid sequence of a protein, estimate its primary location within the cell:	Based on the provided amino acid sequence, it appears that the protein is a membrane protein, likely localized to the endoplasmic reticulum, Golgi apparatus, and/or plasma membrane.The presence of multiple transmembrane domains and the distribution of hydrophobic and hydrophilic residues suggest that this protein is an integral membrane protein.Additionally, the sequence contains several potential phosphorylation sites and potential N-glycosylation sites, which may be involved in protein-protein interactions and/or post-translational modification.	The protein characterized by the structure and amino acid sequence demonstrates rRNA binding and is implicated in the translation. Its subcellular localization is primarily within the cytosol.	Upon analyzing the given protein structure and sequence, it appears that the protein is likely to be **localized in the cytosol compartment of the cell**.
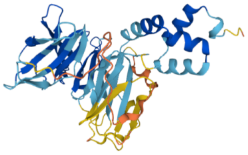 **[O64597]**Please predict any recognizable motifs or domains that this protein might contain. The protein sequence is provided below:	After analyzing the protein sequence, I predict that it contains the following motifs or domains: 1. **Protein kinase C (PKC) consensus sequence**: The sequence contains a motif similar to the PKC consensus sequence (S/T-X-K/R), which is a common recognition site for protein kinase C.	The computational analysis of the structure and sequence suggests the presence of the following protein domains or motifs: F-box	Upon evaluating your submitted structure and sequence, our predictive algorithms **suggest the presence of: F-box**.

Table 2: More case study. The bold font represents the key points of the answers, the blue font represents the correct answers of the key points, and the red font represents the incorrect answers of the key points.

Inputs	Trained with sequences	Prot2Chat	Ground Truth
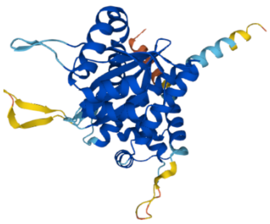 **[O70075]**Assess the function of the protein and its possible involvement in a biological process, based on the protein structure and the following amino acid sequence:	Based on the provided amino acid sequence, the protein is predicted to be a transmembrane protein, likely involved in the protein localization, protein transport, and vesicle-mediated transport.The protein structure suggests a possible role in the regulation of protein localization, vesicle-mediated transport, and protein transport. The sequence contains several features that are indicative of a transmembrane protein, including: 1. A hydrophobic region in the middle of the sequence, which is likely to be the transmembrane domain.	The protein structure and sequence under consideration appears to have a primary function of DNA binding, tRNA binding, and it may be associated with the DNA transposase activity.	The protein structure and sequence under consideration appears to have a primary function of **DNA binding, transposase activity, and it may be associated with the DNA transposition**.
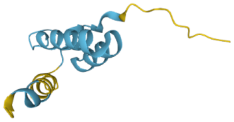 **[P0A179]**Given the protein structure and sequence provided, determine the cellular compartment where it is most likely to be localized: Examine the given protein and share a brief overview of its attributes.	After analyzing the provided protein sequence, here's a brief overview of its attributes: 1. **Length**: The protein sequence has 120 amino acids. 2. **Composition**: The sequence is primarily composed of hydrophilic amino acids, with a slight bias towards hydrophobic amino acids.3. **Motifs**: No obvious motifs or domains are apparent from the sequence. 4. **Charg**: The sequence has a neutral charge, with a slight bias towards basic residues. 5. **Hydrophobicity**: The sequence has a moderate hydrophobicity, with a slight bias towards hydrophobic regions.	A summary of the protein's main attributes with the input structure and amino acid sequence reveals: could be involved in insertion of integral membrane proteins into the membrane.	Based on the provided protein structure and sequence, the predicted subcellular **localization is plasma membrane**.An outline of the key aspects of the protein with the corresponding structure and amino acid sequence is as follows: could be **involved in insertion of integral membrane proteins into the membrane**.

References

Dauparas, J., Anishchenko, I., Bennett, N., Bai, H., Ragotte, R. J., Milles, L. F., Wicky, B. I., Courbet, A., de Haas, R. J., Bethel, N., et al. (2022). Robust deep learning–based protein sequence design using proteinmpnn. *Science*, 378(6615):49–56.

Lin, C.-Y. (2004). ROUGE: A package for automatic evaluation of summaries. In *Text Summarization Branches Out*, pages 74–81, Barcelona, Spain. Association for Computational Linguistics.

Papineni, K., Roukos, S., Ward, T., and Zhu, W.-J. (2002). Bleu: a method for automatic evaluation of machine translation. In Isabelle, P., Charniak, E., and Lin, D., editors, *Proceedings of the 40th Annual Meeting of the Association for Computational Linguistics*, pages 311–318, Philadelphia, Pennsylvania, USA. Association for Computational Linguistics.

Qin, R., Li, Z., He, W., Zhang, M., Wu, Y., Zheng, W., and Xu, X. (2024). Mooncake: A kvcache-centric disaggregated architecture for llm serving. *arXiv preprint arXiv:2407.00079*.
